# Retracted: Effect of Baogong Zhixue Granules Combined with Tranexamic Acid Injection on the Hemodynamics and Reproductive System in Patients with Postpartum Hemorrhage after Cesarean Section

**DOI:** 10.1155/2022/9768545

**Published:** 2022-11-29

**Authors:** Evidence-Based Complementary and Alternative Medicine

*Evidence-Based Complementary and Alternative Medicine* has retracted the article titled “Effect of Baogong Zhixue Granules Combined with Tranexamic Acid Injection on the Hemodynamics and Reproductive System in Patients with Postpartum Hemorrhage after Cesarean Section” [1] due to concerns that the peer review process has been compromised.

Following an investigation conducted by the Hindawi Research Integrity team [2], significant concerns were identified with the peer reviewers assigned to this article; the investigation has concluded that the peer review process was compromised. We therefore can no longer trust the peer review process, and the article is being retracted with the agreement of the Chief Editor.

## References

[1] Cui X., Ji Y., Zhang L., Lv Q., Liu L. (2022). Effect of Baogong Zhixue Granules Combined with Tranexamic Acid Injection on the Hemodynamics and Reproductive System in Patients with Postpartum Hemorrhage after Cesarean Section. *Evidence-Based Complementary and Alternative Medicine*.

[2] Ferguson L. (2022). Advancing Research Integrity Collaboratively and with Vigour. https://www.hindawi.com/post/advancing-research-integrity-collaboratively-and-vigour/.

